# Salivary cortisol and alpha-amylase levels during an assessment procedure correlate differently with risk-taking measures in male and female police recruits

**DOI:** 10.3389/fnbeh.2013.00219

**Published:** 2014-01-16

**Authors:** Ruud van den Bos, Ruben Taris, Bianca Scheppink, Lydia de Haan, Joris C. Verster

**Affiliations:** ^1^Department of Organismal Animal Physiology, Radboud University NijmegenNijmegen, Netherlands; ^2^Police Academy, Recruitment and SelectionApeldoorn, Netherlands; ^3^Division of Pharmacology, Utrecht Institute for Pharmaceutical Sciences, Utrecht UniversityUtrecht, Netherlands; ^4^Centre for Human Psychopharmacology, Swinburne University of TechnologyMelbourne, Australia

**Keywords:** cortisol, alpha-amylase, decision-making, Cambridge Gambling Task, sex, humans

## Abstract

Recent laboratory studies have shown that men display more risk-taking behavior in decision-making tasks following stress, whilst women are more risk-aversive or become more task-focused. In addition, these studies have shown that sex differences are related to levels of the stress hormone cortisol (indicative of activation of the hypothalamus-pituitary-adrenocortical-axis): the higher the levels of cortisol the more risk-taking behavior is shown by men, whereas women generally display more risk-aversive or task-focused behavior following higher levels of cortisol. Here, we assessed whether such relationships hold outside the laboratory, correlating levels of cortisol obtained during a job-related assessment procedure with decision-making parameters in the Cambridge Gambling Task (CGT) in male and female police recruits. The CGT allows for discriminating different aspects of reward-based decision-making. In addition, we correlated levels of alpha-amylase [indicative of activation of the sympatho-adrenomedullary-axis (SAM)] and decision-making parameters. In line with earlier studies men and women only differed in risk-adjustment in the CGT. Salivary cortisol levels correlated positively and strongly with risk-taking measures in men, which was significantly different from the weak negative correlation in women. In contrast, and less strongly so, salivary alpha-amylase levels correlated positively with risk-taking in women, which was significantly different from the weak negative correlation with risk-taking in men. Collectively, these data support and extend data of earlier studies indicating that risky decision-making in men and women is differently affected by stress hormones. The data are briefly discussed in relation to the effects of stress on gambling.

## Introduction

Recently we have reviewed whether sex differences are present in the occurrence and development of disordered gambling (van den Bos et al., 2013a); an area of research still poorly studied (see also van den Bos et al., 2013b). Among others, stress may promote gambling episodes in men and women (Tschibelu and Elman, 2011), and, in addition, may (be expected to) affect gambling behavior as stress has been shown to disrupt reward-based decision-making under laboratory conditions (review: Starcke and Brand, 2012). In particular, studies encompassing both sexes have shown that men display more risk-taking behavior following stress, whilst women are more risk-aversive or become more task-focused (Preston et al., 2007; Lighthall et al., 2009; van den Bos et al., 2009; Mather and Lighthall, 2012). In addition, it has been found that the higher the levels of cortisol [indicative of activation of the hypothalamic-pituitary-adrenal cortex (HPA) axis] the more risk-taking behavior men show (van den Bos et al., 2009), while in general women show more risk-aversive or task-focused behavior (Lighthall et al., 2009; van den Bos et al., 2009). A recent study in men has shown that activation of the sympathetic nervous system [releasing catecholamines, i.e., (nor)adrenaline] is associated with decreased risk-taking, while this study confirmed that cortisol is associated with increased risk-taking (Pabst et al., 2013).

While data in the laboratory using standardized protocols, such as the Trier Social Stress Test, begin to reveal the relationship between sex, neuro-endocrine status and decision-making, they may not be indicative of the effects occurring in real-life, where currently circulating levels of cortisol and catecholamines, related to earlier events, context and time of the day, may determine the outcome of decision-making (see for discussion: van den Bos et al., 2013a,c). Next to understanding the relationship to activities such as gambling, this knowledge may also be of relevance for decision-making behavior in the military, police force, financial business or health care, where decisions often have to be made under highly stressful conditions. When decisions are taken wrongly due to changes in risk-perception under stress they may have a highly negative personal, financial and societal impact (Taylor et al., 2007; LeBlanc et al., 2008; LeBlanc, 2009; Arora et al., 2010; Akinola and Mendes, 2012). Therefore, given the limited body of current knowledge as well as to assess the effects of circulating levels of cortisol and catecholamines on risk-taking, we correlated spontaneously occurring variation in stress hormones during a job-assessment procedure in male and female police recruits with reward-based decision-making parameters in the Cambridge Gambling Task (CGT) (Rogers et al., 1999). Thus, we chose to conduct the study in an applied setting to assess whether laboratory findings would hold under real-life conditions.

The CGT allows for discriminating different aspects of reward-based decision-making, such as risk-taking, impulsivity and risk-adjustment (e.g., Rogers et al., 1999; Deakin et al., 2004; Newcombe et al., 2011; van den Bos et al., 2012). Male and female subjects performed the CGT during their assessment for the Master of Criminal Investigation at the Police Academy. This assessment is generally considered to be stressful by candidates. Thus, rather than using a laboratory set-up with a separate stress group and control group, we used spontaneously occurring variation in levels of salivary cortisol (activation of the HPA-axis; review: Foley and Kirschbaum, 2010) and alpha-amylase [activation of the sympatho-adrenomedullary (SAM) axis; review: Nater and Rohleder, 2009] to correlate physiological changes and behavior. We predicted that the higher the current levels of salivary cortisol in men, the more risk-taking behavior they display, while in women the opposite effect was expected (conform Lighthall et al., 2009; van den Bos et al., 2009). As no data exist regarding sex differences for current salivary alpha-amylase levels and risk-taking behavior, no specific predictions were made for these correlations.

## Materials and methods

### Subjects and procedure

Physically and psychologically healthy men [*n* = 49; age (mean ± *SD*): 28.5 ± 5.4 years; range 22–43 years] and women (*n* = 34; age: 26.7 ± 4.1; range 22–37 years; Student *t*-test; *t* = 1.516, *df* = 81, *p* = 0.133) were recruited from subjects who applied for the Master of Criminal Investigation. All subjects signed an informed consent before participating in this study. The study was performed in accordance with the ethical standards as formulated in the 1964 Declaration of Helsinki [*The Code of Ethics of the World Medical Association (Declaration of Helsinki) for experiments involving humans* http://www.wma.net/en/30publications/10policies/b3/index.html].

Candidates were subjected to a two-day assessment at the Police Academy (Apeldoorn, Netherlands) containing a series of physical tests (day 1) and psychological tests (day 2). Only candidates who passed the physical tests enrolled into the second day of psychological tests. The psychological tests encompassed cognitive ability tests, a personality inventory, a psychological interview and a job-related simulation [Fact Finding Decision-Making (FFDM) task]. For logistic reasons inherent to the assessment procedure at the Police Academy the order of tests varied between subjects. Therefore, we scheduled the CGT to follow the FFDM task for each candidate, such that each candidate had the same test immediately before the CGT.

To determine daytime cortisol and alpha-amylase levels in saliva, samples using Salivettes® Cortisol (Sarstedt, Nümbrecht, Germany) were collected at four moments during the assessment procedure according to procedures and recommendations of the manufacturer: (1) when subjects arrived early in the morning (8:15–8.45 AM), (2) directly before the start of the FFDM task (8:45 AM, 10:15 AM, or 2:15 PM), (3) after the FFDM, which lasted 1.45 h, which is directly *before* the CGT (10:30 AM, 0:15 PM, or 4:00 PM), and (4) *after* the CGT (11.00 AM, 1:00 PM, 4.30 PM; see below). In cases where subjects started with the FFDM task as their first assignment of the day saliva sample 1 and 2 collided. As only levels *before* (3) and *after* (4) the CGT are of relevance for the present paper, only these values will be reported here. We chose to obtain levels of salivary cortisol and alpha-amylase *before* and *after* the CGT to optimize correlations between these levels and task-performance. It should be noted that the CGT by itself is not a stress-inducing task.

### Cambridge gambling task

The CGT was developed to assess different aspects of decision-making (Rogers et al., 1999). Detailed information on the task and procedure can be found in the manual of CGT (www.cantab.com) and earlier published papers (Rogers et al., 1999; Deakin et al., 2004; Newcombe et al., 2011; van den Bos et al., 2012). In brief, in each trial the subject is presented with an array of 10 red and blue boxes. The subject must guess if a yellow token is hidden in a red or blue box by touching one of two rectangles, with the word “red” or “blue,” on the screen. The ratio of red to blue boxes varies from trial to trial. Some trials have highly favorable odds (e.g., nine blue boxes/one red box), while others have less favorable odds (e.g., six blue boxes/four red boxes). In the gambling stages the subjects start with 100 points. Subjects can select a proportion of these points (5, 25, 50, 75, or 95%), displayed in an ascending or a descending order, to bet on whether the yellow token is hidden in a blue or red box. In the ascending order subjects start with the option to gamble 5% of their credit points on their choice (blue or red) after which percentages increase (as indicated above; about 2 s delay between options) until subjects press the button on the screen, which is the taken as their choice for this trial. In the descending order subjects start with the option to gamble 95% of their credit points on their choice (blue or red) after which percentages decrease (as indicated above; about 2 s delay between options) until subjects press the button on the screen, which is the taken as their choice for this trial.

The task contains five stages. The first stage is a decision-making stage. Subjects have to choose whether the token is hidden in a blue or red box (four trials). The second stage is a gambling training stage (ascending order; four trials). Subjects have to choose whether the token is hidden in a blue or red box and then select the amount they wish to bet, both by touching the screen. The third stage is a gambling test stage (ascending order; four series of nine trials). The fourth stage is a gambling training stage (descending order; four trials). The fifth stage is a gambling test stage (descending order; four series of nine trials). The subjects must try to accumulate as many points as possible. Whether subjects start with the ascending order followed by the descending order or the other way round is randomized across test-subjects. The task takes 20–25 min to complete.

The following measures are extracted: (1) *Quality of decision-making (QDM)*: a measure which reflects the ability of subjects to judge the likelihood of events to happen (cognition), i.e., it measures the proportion of trials on which the subject chose to gamble on the more likely outcome. The higher the value the more appropriate subjects behave according to the situation. (2) *Overall proportion bet (OPB)* and *Risk taking (Likely Proportion Bet; LPB)*: both parameters are measures of risk tolerance, i.e., the higher the value the more subjects tolerate risks. *OPB* measures the average proportion of the current points total that the subject chose to risk on each gamble test trial, including trials on which they bet on the less likely outcome. However, differences may exist regarding betting behavior on likely or unlikely options. For instance, subjects may bet a lower amount of credit points when choosing an unlikely option than a likely option. Therefore, the CGT also includes a second parameter, which is labeled *Risk taking* in the manual, but will be labeled *LPB* here to stay in line with the previous parameter. This measure reports the mean proportion of the current points total that the subject chose to risk on gamble test trials for which they had chosen the more likely outcome, i.e., trials on which they had a higher chance of winning than losing. *OPB* equals *LPB* when subjects hardly choose the unlikely option, i.e., in such case they are highly correlated (van den Bos et al., 2012). In line with our earlier studies (van den Bos et al., 2012) we used both measures. (3) *Deliberation time (DT)* and *Delay Aversion (DA)*: two measures which may reflect impulsivity. *DT* is the mean latency from presentation of the colored boxes to the subject's choice of which color to bet on. The higher the value the longer subjects take to decide. This parameter measures reflection impulsivity although the CGT is not a task in which delay increases the information available. Subjects who are unable/unwilling to wait will bet larger amounts when they are presented in descending order than in ascending order. This is reflected in *DA*, which is calculated as the difference between the risk-taking score in the descend condition and the ascend condition. This measure reflects DA, but may also reflect motor impulsivity. The higher the value the more impulsive subjects are or the more they avoid delays. (4) *Risk adjustment (RA)*: the ability to adjust betting behavior according to the likelihood of winning (interaction cognition-reward), i.e., subjects will gamble more of their current points when the odds are strongly in favor of them. A low RA score could be interpreted as a failure to use the available information when making a decision. This measure reflects the tendency to bet a higher proportion of points on trials when the large majority of the boxes are of the color chosen (e.g., 9:1) than when a small majority of the boxes are of the color chosen (e.g., 6:4). This RA score was calculated as the degree to which the risk differed across the ratios, as a proportion of the overall amount risked by that subject: RA = [2^*^(% bet at 9:1) + (% bet at 8:2) − (% bet at 7:3) − 2^*^(% bet at 6:4)]/average % bet. A RA score of approximately zero reflects no systematic tendency to take differential risks across the ratios, whereas a high positive score indicates a tendency to bet a larger proportion of the available points on the higher ratio (9:1 and 8:2) trials than on the lower ratio (7:3 and 6:4) trials.

### Physiological measurements

Saliva samples were stored at −20°C directly following collection and remained at this temperature for a maximum period of 4 months until processing at the Specieel Laboratorium Endocrinologie (UMCU, Utrecht, Netherlands).

Cortisol in saliva was measured without extraction using an in house competitive radio-immunoassay employing a polyclonal anticortisol-antibody (K7348). [1,2-^3^H(N)]-Hydrocortisone (PerkinElmer NET396250UC) was used as a tracer. The lower limit of detection was 1.0 nmol/l and inter-assay variation was <6% at 4-29 nmol/l (*n* = 33). Intra-assay variation was <4% (*n* = 10). Samples with levels >100 nmol/L were diluted 10× with assay buffer.

Alpha-amylase in saliva was measured on a Beckman-Coulter AU5811 chemistry analyzer (Beckman-Coulter Inc., Brea, CA). Saliva samples were diluted 1000× with 0.2% BSA in 0.01 M phosphate buffer pH 7.0. Interassay variation was 3,6% at 200.000 U/L (*n* = 10).

Although cortisol and alpha-amylase levels may differ between women which use oral contraceptives or not, and cortisol levels vary across the menstrual cycle (Foley and Kirschbaum, 2010) we did not take these differences into account here as we were interested in the effects of the current levels of cortisol and alpha-amylase on decision-making behavior (see also van den Bos et al., 2009; de Visser et al., 2010). However, the number of male and female subjects was counterbalanced across morning and afternoon periods to account for differences in morning and afternoon values (Nater et al., 2007).

### Statistical analysis

All statistical analyses were carried out using SPSS 16.0 for Windows or the Vasserstats website (www.vasserstats.net) where needed. Tests are indicated in the Results section. Significance (two-tailed) was set at *p* ≤ 0.05; *p*-values > 0.05 and ≤ 0.10 were considered trends, while *p*-values > 0.10 were considered non-significant (NS).

## Results

### Cambridge gambling task

No differences were found between men and women for choosing the most likely option [QDM: men vs. women (mean ± *SD*): 0.96 ± 0.06 vs. 0.95 ± 0.06; Student *t*-test, NS], for risk-taking measures [OPB: 0.53 ± 0.09 vs. 0.54 ± 0.11 (Student *t*-test, NS); LPB: 0.58 ± 0.10 vs. 0.58 ± 0.11 (Student *t*-test, NS)] and for impulsivity measures [DT: 2019.6 ± 1132.8 ms vs. 1749.8 ± 565.2 ms (Student *t*-test, NS); DA: 0.14 ± 0.12 vs. 0.19 ± 0.16 (Student *t*-test, NS)]. Only risk-adjustment differed significantly between men and women (1.82 ± 0.80 vs. 1.46 ± 0.74; Student *t*-test: *t* = 2.098, *df* = 81, *p* = 0.039). As subjects chose the most likely option often (QDM > 0.95) it should be noted that *OPB* and *LPB* are virtually identical. These measures were strongly correlated in men and women: men: *r* = 0.975, *n* = 49, *p* < 0.001; women: *r* = 0.979, *n* = 34, *p* < 0.001.

### Salivary cortisol and alpha-amylase

Table 1A shows the levels of salivary cortisol and alpha-amylase *before* the CGT at the different time-points across the day, while Table 1B shows the levels of salivary cortisol and alpha-amylase *after* the CGT at the different time-points across the day. While cortisol levels decreased across time-points in both cases [*before*: two-way ANOVA; time-points: *F*_(2, 77)_ = 6.552, *p* = 0.002; *after*: *F*_(2, 77)_ = 6.345, *p* = 0.003], no differences were found between men and women [*before*: sex: *F*_(1, 77)_ = 0.801, NS; sex^*^time-points: *F*_(2, 77)_ = 0.612, NS; *after*: sex: *F*_(1, 77)_ = 0.011, NS; sex^*^time-points: *F*_(2, 77)_ = 1.186, NS]. In both cases no differences were observed for time-points or sex for alpha-amylase levels (*before*: *F* values <0.671, *p*-values >0.415; *after*: *F* values <1.566, *p*-values >0.215).

**Table 1A:** **Salivary cortisol and alpha-amylase levels (mean ± *SD*) *before* the CGT in men and women at different time-points during the day; number of subjects is indicated between brackets**.

	**Men**	**0:15 PM**	**4:00 PM**	**Women**	**0:15 PM**	**4:00 PM**
	**10:30 AM**			**10:30 AM**		
Cortisol	19.4 ± 7.7	18.8 ± 6.3	13.0 ± 4.3	18.5 ± 6.5	15.4 ± 4.5	13.6 ± 4.6
(nmol/l)	(17)	(16)	(16)	(16)	(7)	(11)
Alpha Amylase	457.117 ± 276.638	454.125 ± 332.008	457.250 ± 288.402	369.250 ± 224.139	457.142 ± 367.517	383.636 ± 184.590
(U/l)	(17)	(16)	(16)	(16)	(7)	(11)

**Table 1B:** **Salivary cortisol and alpha-amylase levels (mean ± *SD*) *after* the CGT in men and women at different time-points during the day; number of subjects is indicated between brackets**.

	**Men**	**1:00 PM**	**4:30 PM**	**Women**	**1:00 PM**	**4:30 PM**
	**11:00 AM**			**11:00 AM**		
Cortisol	15.8 ± 6.6	15.2 ± 5.4	10.5 ± 2.9	16.3 ± 5.0	12.6 ± 2.5	12.4 ± 4.0
(nmol/l)	(17)	(16)	(16)	(16)	(7)	(11)
Alpha Amylase	316.529 ± 179.901	338.825 ± 264.301	306.875 ± 170.377	241.812 ± 162.416	296.285 ± 234.909	255.090 ± 133.498
(U/l)	(17)	(16)	(16)	(16)	(7)	(11)

### Correlation between CGT parameters and salivary cortisol as well as alpha-amylase

In both men and women cortisol as well as alpha-amylase levels *before* and *after* the CGT were highly correlated: men, cortisol: *r* = 0.971, *n* = 49, *p* < 0.001; women, cortisol: *r* = 0.953, *n* = 34, *p* < 0.001; men, alpha-amylase: *r* = 0.716, *n* = 49, *p* < 0.001; women, alpha-amylase: *r* = 0.926, *n* = 34, *p* < 0.001. To reduce the number of correlations we therefore decided to calculate the mean of the levels *before* and *after* the CGT to capture the average levels of salivary cortisol and alpha-amylase *during* the task and correlate these average levels with the CGT parameters.

**Figure 1:**
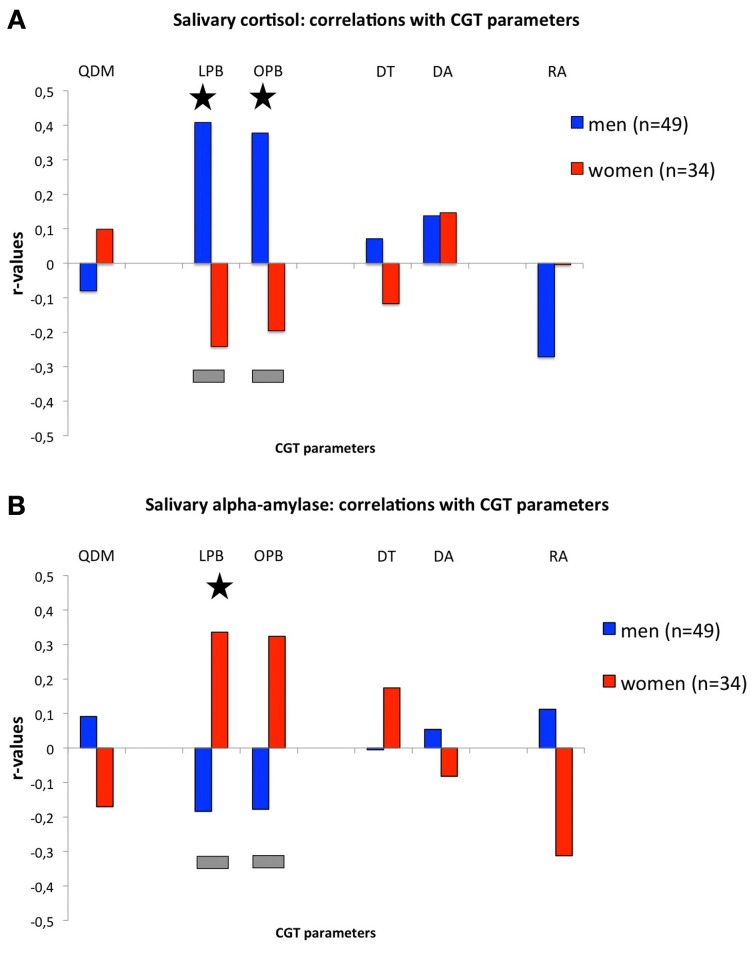
**(A)** Correlations (*r*-values; y-axis) between cortisol levels *during* the CGT and CGT parameters (x-axis). **(B)** Correlations (*r*-values; y-axis) between alpha-amylase levels *during* the CGT and CGT parameters (x-axis). *For both panels*: QDM, quality of decision-making; LPB, likely proportion bet; OPB, overall proportion bet; DT, deliberation time; DA, delay aversion; RA, risk-adjustment. Gray bars indicate significant differences between *r*-values of men and women (see text for details); asterisks indicate significant *r*-values (see text for details).

Figure 1A, shows the correlations between salivary cortisol levels and CGT measures. Salivary cortisol levels were positively and significantly correlated with LPB (*r* = 0.408, *n* = 49, *p* = 0.004) and OPB (*r* = 0.378, *n* = 49, *p* = 0.007) in men, which were significantly different from the negative, but non-significant, correlations in women (LPB: *r* = −0.241, *n* = 34, NS; Fisher-*r*-to-*z*, *z* = 2.92 *p* = 0.004; OPB: *r* = −0.196, *n* = 34, NS; Fisher-*r*-to-*z*, *z* = 2.57, *p* = 0.01). Cortisol levels in men tended to correlate negatively with RA (*r* = −0.271, *n* = 49, *p* = 0.06). No other significant differences or trends were found. It should be noted that the significant correlations in men remain even when we would correct for the number of correlations (*p-value = 0.05/6 = 0.0083)*. In, addition we confirmed that the main effects of LPB and OPB in men were not due to differences in levels of cortisol across time-points *per se* (see Tables 1A,B) as correlations remained significant following correction for differences in time-points: *before* CGT: no correction OPB: *r* = 0.365, *df* = 47, *p* = 0.01, LPB: *r* = 0.395, *df* = 47, *p* = 0.005; with correction (partial correlations): OPB: *r* = 0.287, *df* = 46, *p* = 0.048; LPB: *r* = 0.329, *df* = 46, *p* = 0.023, *after* CGT: no correction: OPB: *r* = 0.387, *df* = 47, *p* = 0.006; LPB: *r* = 0.418, *df* = 47, *p* = 0.003; with correction (partial correlations): OPB: *r* = 0.314, *df* = 46, *p* = 0.030; LPB: *r* = 0.355, *df* = 46, *p* = 0.013.

**Figure 2:**
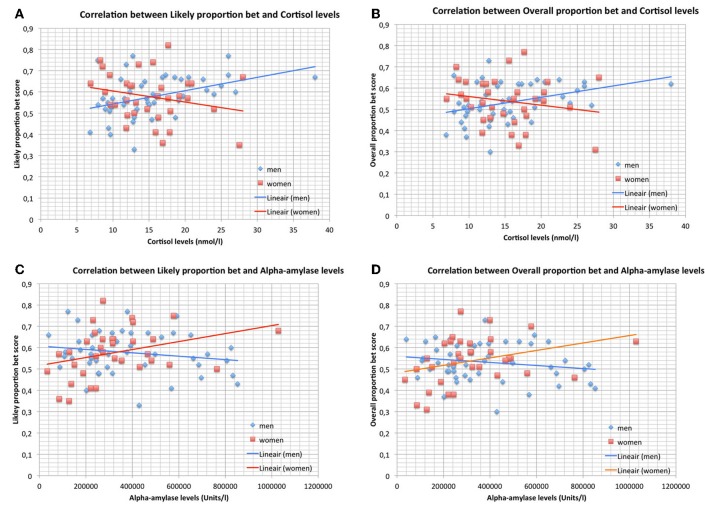
**(A)** Correlation between Likely proportion bet and cortisol levels *during* the CGT in men (*n* = 49) and women (*n* = 34). Trend-lines are added to indicate correlations. **(B)** Correlation between Overall proportion bet and cortisol levels *during* the CGT in men (*n* = 49) and women (*n* = 34). Trend-lines are added to indicate correlations. **(C)** Correlation between Likely proportion bet and alpha-amylase levels *during* the CGT in men (*n* = 49) and women (*n* = 34). Trend-lines are added to indicate correlations. **(D)** Correlation between Overall proportion bet and alpha-amylase levels *during* the CGT in men (*n* = 49) and women (*n* = 34). Trend-lines are added to indicate correlations.

Figures 2A,B, show the significant correlations between salivary cortisol levels and LPB as well as OPB scores in men and the non-significant correlations in women. The panels show that risk-taking measures and cortisol levels were within the same range in men and women. The mean values of cortisol were not different between men and women (men vs. women; mean ± *SD*; nmol/l): 15.50 ± 6.20 vs. 15.24 ± 5.18 (Student *t*-test, NS).

Figure 1B, shows the correlations between salivary alpha-amylase levels and CGT measures. Salivary alpha-amylase levels correlated positively and significantly with LPB (*r* = 0.336, *n* = 34, *p* = 0.05), while a trend was observed for the correlation with OPB (*r* = 0.324, *n* = 34, *p* = 0.06), in women, which were significantly different from the negative, but non-significant, correlations in men (LPB: *r* = −0.184, *n* = 49, NS; Fisher-*r*-to-*z*, *z* = −2.31, *p* = 0.02; OPB: *r* = −0.178, *n* = 49, NS; Fisher-*r*-to-*z*, *z* = −2.22, *p* = 0.03). Risk-adjustment tended to correlate negatively in women (*r* = −0.312, *n* = 34, *p* = 0.07), which tended to differ from the non-significant positive correlation in men (*r* = 0.112, *n* = 49, NS; Fisher *r*-to-*z*, *z* = 1.87, *p* = 0.06). No other significant differences or trends were found. It should be noted that the significant correlations in women disappear when we would correct for the number of correlations (*p*-value = 0.05/6 = 0.0083).

Figures 2C,D, show the significant correlations between salivary alpha-amylase levels and LPB as well as OPB scores in women and the non-significant correlations in men. The panels show that risk-taking measures and alpha-amylase levels were within the same range in men and women. The mean values of alpha-amylase were not different between men and women (men vs. women; mean ± *SD*; U/l): 379.859 ± 219.974 vs. 324.397 ± 201.199 (Student *t*-test, NS).

A significant negative correlation was found between salivary cortisol and alpha-amylase levels in women (*r* = −0.394, *n* = 34, *p* = 0.02); this was not the case in men (*r* = −0.137, *n* = 49, NS). We therefore used multiple regression to assess whether the combination explained more of the variance. This was not case (not shown). Since it was observed earlier that in women curve-linear relationships may exist between cortisol and risk-taking (van den Bos et al., 2009), this possibility was also explored for cortisol and alpha-amylase and LPB as well OPB scores. However, no such curve-linear relationships were found (not shown).

Figures 2A,B, suggest that the risk-taking measures are lower in men than women at the low end of cortisol levels, while the opposite is the case at the high end of cortisol levels. To capture this as well as to further underpin the correlations we calculated the quartiles for the cortisol values and assessed risk-taking measures according to these quartiles. We only compared the low end (quartile 1) and the high end values (quartile 4). Table 2A shows that no difference existed between men and women regarding the cortisol levels when quartiles for men and women were calculated. In contrast risk-taking measures changed differently in men and women related to the low and high end quartiles. While in men LPB and OPB increased significantly from quartile 1 to 4, in women they did not, in line with the correlations reported above. Furthermore, LPB and OPB values in women were higher than values of men at the low end, while the opposite was true at the high end of cortisol quartiles. In addition, alpha-amylase levels tended to be lower at high end of the cortisol levels in men, but not women.

**Table 2A:**
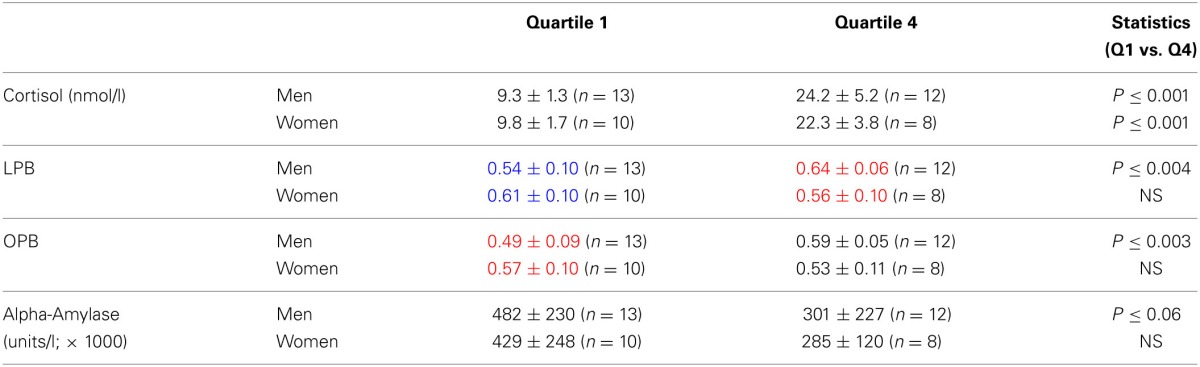
**Risk-taking parameters and salivary alpha-amylase levels (mean ± *SD*) in men and women calculated according to cortisol-related quartiles (see text)**.

*Red: values significantly different between men and women; Blue: values trend between men and women*.

Figures 2C,D, suggest that the risk-taking measures are lower in women than men at low levels of alpha-amylase, while the opposite is the case at high levels. To capture this as well as to further underpin the correlations we calculated the quartiles for the alpha-amylase values and assessed risk-taking measures according to these quartiles. We only compared the low end (quartile 1) and the high end values (quartile 4). Table 2B indicates that women showed overall slightly lower alpha-amylase levels. Risk-taking measures changed differently in men and women related to the low and high end of the quartiles. While in women LPB and OPB increased significantly, in men they did not, in line with the correlations reported above. Furthermore, LPB and OPB values in men were higher than values in women at the low end, while this was not the case at the high end of alpha-amylase levels. In addition, cortisol levels tended to be lower at high end of the alpha-amylase quartiles in women, but not men.

**Table 2B:**
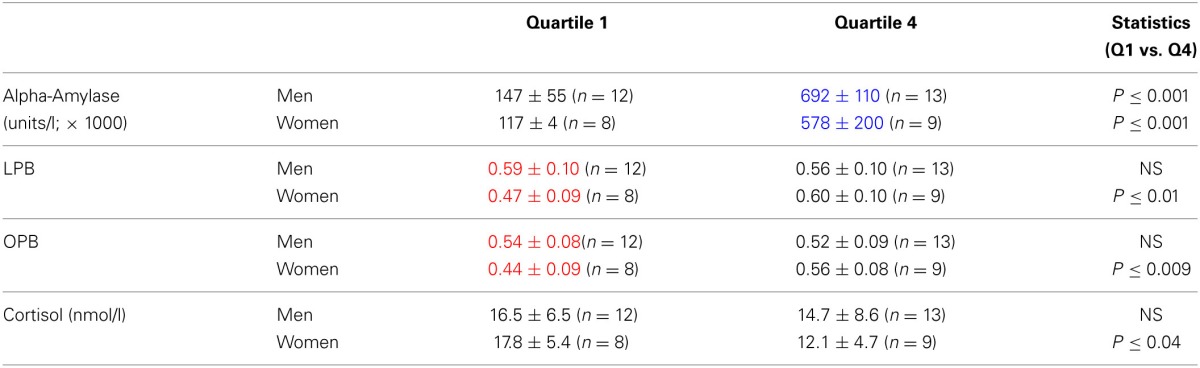
**Risk-taking parameters and salivary cortisol levels (mean ± *SD*) in men and women calculated according to alpha-amylase-related quartiles (see text)**.

*Red: values significantly different between men and women; Blue: values trend between men and women*.

## Discussion

The aim of this study was to determine whether individual differences in current levels of salivary cortisol (activation of the HPA-axis) and/or alpha-amylase (activation of the SAM-axis) in an assessment procedure were related to differences in decision-making related parameters in the CGT in men and women. The main findings of this study were that, (1) men and women differed in risk-adjustment in the CGT, (2) cortisol levels correlated strongly positively with risk-taking measures in men, which was significantly different from the weak negative correlation in women, and (3) alpha-amylase levels correlated positively, but not strongly, with risk-taking in women, which was significantly different from the weak negative correlation with risk-taking in men. Collectively, these data support and extend data of earlier studies indicating that risky decision-making in men and women is differently affected by stress hormones (Lighthall et al., 2009; van den Bos et al., 2009).

### General

Men and women only differed in risk-adjustment in the CGT. This difference between sexes matches the outcome of earlier studies (Deakin et al., 2004; van den Bos et al., 2012), indicating that this is a robust finding between sexes regarding decision-making (review: van den Bos et al., 2013b,c). As we did not include a control group we cannot address the question whether CGT parameters, for instance those related to risk-taking, were in generally higher or lower in the job assessment group. However, earlier data of a group of subjects within the same age range (van den Bos et al., 2012) suggest that LPB and OPB scores were overall higher in the present study.

We did not assess levels of (psychological or subjective) stress experienced by our test-subjects, as this was not the objective of this study. However, the assessment procedure is generally considered to be stressful by the candidates. As increased levels of subjective stress and increased levels of stress hormones co-occur (e.g., Starcke and Brand, 2012; van den Bos et al., 2013c), the levels of salivary cortisol and alpha-amylase, that we observed here, suggest that subjects may have been psychologically stressed: levels were above for what may normally be found across the day (e.g., Nater et al., 2007; Nater and Rohleder, 2009; van den Bos et al., 2009; de Visser et al., 2010). Therefore, discussions which follow should be considered against the background of possibly psychologically stressed subjects.

### CGT, cortisol, and alpha-amylase

A striking finding was that while risk-taking measures and current salivary cortisol levels during the assessment procedure were not different between men and women, current salivary cortisol levels were strongly and positively correlated with risk-taking measures in men, which was significantly different from the non-significant negative correlation between current salivary cortisol levels and risk-taking parameters in women. These correlations and differences between sexes were supported by the analysis of differences in risk-taking parameters related to the low and high end of cortisol quartiles. In conjunction with the trend for a negative correlation with risk-adjustment the data in men suggest that related to HPA-axis activation men increase their bets across the entire range of odd-ratio's without adjusting betting behavior according to the odds of winning. This increased risk-taking may be related to a cortisol induced increase in reward-processing and decrease in punishment-processing (Putman et al., 2010; Mather and Lighthall, 2012).

An obvious limitation of our study is that we did not explicitly use a control and stress group as in laboratory studies to manipulate cortisol levels (Lighthall et al., 2009; van den Bos et al., 2009). Still, our data are in line with data obtained in the laboratory, where it has been shown, using a stress and control group, that higher levels of salivary cortisol are associated with higher levels of risk-taking behavior in men and higher levels of salivary cortisol with risk-aversive and/or task-focused behavior in women (Lighthall et al., 2009; van den Bos et al., 2009; Pabst et al., 2013). Thus, this study confirms and extends earlier reports and points to a general difference between sexes. Furthermore, these data add to the validity of laboratory studies showing that differences in cortisol levels in daily life affect the behavior of men and women differently. In contrast to an earlier study (van den Bos et al., 2009) we did not observe a curve-linear relationship between cortisol and task-performance in women. This may be related to differences between the (parameters of) CGT and Iowa Gambling Task or the way stress was elicited (short-lasting Trier Social Stress Test vs. long-lasting assessment procedure).

A second striking finding, but less strongly than the first, was that while current salivary alpha-amylase levels were not different between men and women, current salivary alpha-amylase levels were differently correlated with risk-taking measures in men and women: salivary alpha-amylase levels correlated positively with risk-taking in women, which was significantly different from the non-significant negative correlations with risk-taking in men. These correlations and differences between sexes were supported by the analysis of differences in risk-taking parameters related to the low and high end alpha-amylase quartiles. In conjunction with the trend for a negative correlation with risk-adjustment the data in women suggest that related to SAM-axis activation women increase their bets across the entire range of odd-ratio's without adjusting betting behavior according to the odds of winning. Although measuring salivary alpha-amylase may be indicative of SAM-axis activation (Nater and Rohleder, 2009; but see Bosch et al., 2011 for critical remarks) the present results should be confirmed using other parameters indicative of SAM-axis activation such as heart rate and heart rate variability.

A recent study in men showed that an increase in SAM-axis activation was associated with a decrease in risk-taking behavior (Pabst et al., 2013). While we did not observe a clear-cut relation between SAM-axis activation and risk-taking here in men, the sign of the correlation was in the same direction as in the study by Pabst et al. (2013). Currently, no studies have studied SAM-axis activation regarding reward-based decision-making in both men and women. These data thus await further confirmation in laboratory studies. However, one recent study clearly showed a difference between men and women regarding amygdala activation, emotional memory and noradrenaline (Schwabe et al., 2013) hinting at differences between men and women in the way SAM-axis activation may affect behavior.

It would be tempting to suggest from the present data that in men low levels of cortisol (low HPA-axis activation) and high levels of alpha-amylase (high SAM-axis activation) are associated with lower risk-taking levels than in women, while the opposite is the case for high levels of cortisol and low levels of alpha-amylase. Similarly, it would be tempting to suggest that in women low levels of cortisol (low HPA-axis activation) and high levels of alpha-amylase (high SAM-axis activation) are associated with higher risk-taking levels than in men, while the opposite is the case for high levels of cortisol and low levels of alpha-amylase. While we observed an inverse relationship between cortisol and alpha-amylase in women, the relationship in men was less strong and clear, although the analysis using quartiles did suggest such an inverse relationship. At present therefore this precludes drawing too strong conclusions regarding the interplay of HPA-axis and SAM-axis activation as well as the role of differences in coping styles in men and women [see for a discussion van den Bos et al. (2013c)]. Thus, while the data do not allow for extensive speculation as yet, they do suggest differences in the effects of SAM-axis and HPA-axis activation on risk-taking behavior in men and women. Future studies should focus on differences in the interaction between HPA-axis and SAM-axis activation in men and women in more detail.

The present study clearly extends data of previous studies further as the CGT measures also other aspects of decision-making. Thus, we did not observe any correlation between cortisol levels or alpha-amylase levels with other measures of decision-making such as impulsivity as measured by *DT* (speed of decisions; reflective impulsivity) and delay-aversion (the inability to wait, motor impulsivity) and the ability to assess whether events are more or less likely to happen (QDM; cognition). It has been suggested that acute stress may increase the speed with which subjects make choices, indicative of a loss of top-down control (Keinan et al., 1987; Porcelli and Delgado, 2009). While we did observe that stress increased decision-making speed in women in our earlier study (van den Bos et al., 2009), this effect was independent of cortisol levels. In a delay discounting task, which measures aspects of impulsivity or levels of self-control it was shown that low levels of saliva alpha-amylase correlate with high levels of impulsivity in men (Takahashi et al., 2007). These data seem in line with the weak correlation between alpha-amylase levels and risk-taking in men that we observed here. In another study it was shown that high and low impulsive male subjects did not differ in basal or gambling induced increases in cortisol levels (Krueger et al., 2005), suggesting no direct relationship between impulsivity and cortisol, which is in line with the data observed here. Future studies should further examine the relationship between speed of decision-making, different forms of impulsivity and stress in more detail.

### Neuronal underpinnings

As to the underlying neural substrates, sex differences in the regulation of the balance between prefrontal areas and subcortical areas may underlie behavioral differences as we have recently discussed extensively elsewhere (van den Bos et al., 2013c; see also Wang et al., 2007). We refer therefore to this review for detailed information. Here, we only allude to general conclusions, especially related to the effects of cortisol as this has been studied in more detail than adrenergic effects (Schwabe et al., 2013). The increase in risk-taking behavior in men in reward-related decision-making under high levels of cortisol may be associated with a loss of top-down control of prefrontal (lateral orbitofrontal cortex and dorsolateral prefrontal cortex) over subcortical structures. Furthermore, within the limbic system high levels of cortisol may shift the balance of the activity of the ventral striatum (reward-related behavior) and amygdala (punishment-related behavior) toward the ventral striatum. In line with this, it was recently observed that systemic injections of corticosterone in male rats in a rodent analog of the Iowa Gambling Task disrupted decision-making performance, which was associated with changes in activity in prefrontal structures (Koot et al., 2013). As to the underlying neural substrate in women it seems that top-down control may actually be increased under stress, related to levels of cortisol, with among others a lower striatal and a stronger amygdala activity. It has been suggested that the persistent activity in, for instance, the anterior cingulate cortex following a stressful experience in women may be associated with the development of depressive symptoms in women related to tendencies of ruminative thinking. The menstrual cycle has a strong effect on the outcome of stress-related changes in neuronal activity (Goldstein et al., 2010; Ter Horst et al., 2013). At present changes in neuronal activity in women are less clear and straightforward than in men. However, by and large these changes in women seem compatible with a shift toward risk-aversive behavior. However, given the current lack of studies that have assessed the behavior of women in decision-making tasks, changes in decision-making behavior are better documented in men than women. Clearly, there is a need for more studies measuring stress, stress hormones and decision-making behavior in men and women under the same conditions using fMRI to assess task-related changes in neuronal activity (Lighthall et al., 2011; Mather and Lighthall, 2012; Porcelli et al., 2012).

### Implications

The data of this study add to the growing number of studies showing differences between men and women in task-performance encompassing emotional regulation (Cahill, 2006; van den Bos et al., 2012, 2013a,b,c). Related to gambling we have elsewhere discussed that more attention should be given to assessing sex differences in the tendency to engage in gambling and develop disordered gambling (van den Bos et al., 2013a). While stress may trigger gambling episodes, underlying reasons for this may be different, e.g., excitement in men vs. overcoming negative mood in women (van den Bos et al., 2013a). In addition, here we show that depending on neuro-endocrine status the consequences in men and women may be different when being involved in gambling episodes. It is clear that studies are needed to assess whether these neuro-endocrine differences also relate to patterns of problematic gambling behavior in real-life.

Finally, the data suggest that some individuals in the military, police force, financial business or health care, which may experience high levels of work-related stress throughout the day, may be at risk of taking wrong decisions due to strong HPA-axis and/or SAM-axis induced changes in risk-perception (Taylor et al., 2007; LeBlanc et al., 2008; LeBlanc, 2009; Arora et al., 2010; Akinola and Mendes, 2012). Both high tendencies to take risks and high tendencies to avoid them may not be optimal for job fulfillment (van den Bos et al., 2013c). Given that police officers may have to take decisions at unexpected time-points during a potential stressful day, the design of the study mimics this situation. Laboratory conditions may not adequately address such a dynamic situation. By doing so, our study revealed differences in patterns between men and women due to (long-term) activation of the HPA-axis and SAM-axis. These data may in turn lead to new laboratory designs for testing the effects of stress on decision-making.

## Conclusion

In conclusion, the data of this study show that high levels of HPA-axis and SAM-axis activation may have different effects in men and women on risk-taking behavior. Future studies should concentrate on the underlying mechanisms of these sex differences.

## Author contributions

Ruud van den Bos, Ruben Taris, Lydia de Haan, Joris C. Verster, and Bianca Scheppink designed the experiment. Bianca Scheppink and Ruben Taris conducted the research. Bianca Scheppink, Ruben Taris, and Ruud van den Bos analyzed the data. Ruud van den Bos, Ruben Taris, Bianca Scheppink, Lydia de Haan, and Joris C. Verster wrote the manuscript.

### Conflict of interest statement

Joris C. Verster has received research support from Takeda Pharmaceuticals, Red Bull GmbH, and acted as consultant for Sanofi-Aventis, Transcept, Takeda, Sepracor, Red Bull GmbH, Deenox, Trimbos Institute, and CBD. Ruud van den Bos acts as consultant for Chardon Pharma. The other authors declare that the research was conducted in the absence of any commercial or financial relationships that could be construed as a potential conflict of interest.

## References

[B1] Akinola M. Mendes W. B. (2012). Stress-induced cortisol facilitates threat-related decision making among police officers. Behav. Neurosci. 126, 167–174 10.1037/a0026657 22141468

[B2] Arora S. Sevdalis N. Nestel D. Woloshynowych M. Darzi A. Kneebone R. (2010). The impact of stress on surgical performance: a systematic review of the literature. Surgery 147, 318–330 10.1016/j.surg.2009.10.007 20004924

[B3] Bosch J. A. Veerman E. C. I. de Geus E. J. Proctor G. B. (2011). α-Amylase as a reliable and convenient measure of sympathetic activity: don't start salivating just yet! Psychoneuroendocrinology 36, 449–453 10.1016/j.psyneuen.2010.12.019 21295411

[B4] Cahill L. (2006).Why sex matter for neuroscience. Nat. Rev. Neurosci. 7, 477–484 10.1038/nrn1909 16688123

[B5] Deakin J. Aitken M. Robbins T. Sahakian B. J. (2004). Risk taking during decision-making in normal volunteers changes with age. J. Int. Neuropsychol. Soc. 10, 590–598 10.1017/S1355617704104104 15327737

[B6] de Visser L. van der Knaap L. J. van de Loo A. J. A. E. van der Weerd C. M. M. Ohl F. van den Bos R. (2010). Trait anxiety affects decision-making differently in healthy men and women: towards gender-specific endophenotypes of anxiety. Neuropsychologia 48, 1598–1606 10.1016/j.neuropsychologia.2010.01.027 20138896

[B7] Foley P. Kirschbaum C. (2010). Human hypothalamus-pituitary- adrenal axis responses to acute psychosocial stress in laboratory settings. Neurosci. Biobehav. Rev. 35, 91–96 10.1016/j.neubiorev.2010.01.010 20109491

[B8] Goldstein J. M. Jerram M. Abbs B. Whitfield-Gabrieli S. Makris N. (2010). Sex differences in stress response circuitry activation dependent on female hormonal cycle. J. Neurosci. 30, 431–438 10.1523/JNEUROSCI.3021-09.2010 20071507 PMC2827936

[B9] Keinan G. Friedland N. Ben-Porath Y. (1987). Decision making under stress: scanning of alternatives under physical threat. Acta Psychol. 64, 219–228 10.1016/0001-6918(87)90008-43572731

[B10] Koot S. Baars A. Hesseling P. van den Bos R. Joëls M. (2013) Time-dependent effects of corticosterone on reward-based decision-making in a rodent model of the Iowa Gambling Task. Neuropharmacology 70, 306–315 10.1016/j.neuropharm.2013.02.008 23474014

[B11] Krueger T. H. C. Schedlowski M. Meyer G. (2005). Cortisol and heart rate measures during casino gambling in relation to impulsivity. Neuropsychobiology 52, 206–211 10.1159/000089004 16244502

[B12] LeBlanc V. R. (2009). The effects of acute stress on performance: implications for health professions education. Acad. Med. 84(10 Suppl.), S25–S33 10.1097/ACM.0b013e3181b37b8f 19907380

[B13] LeBlanc V. R. Regehr C. Jelley R. B. Barath I. (2008). The relationship between coping styles, performance, and responses to stressful scenarios in police recruits. Int. J. Stress Manage. 15, 76–93 10.1037/1072-5245.15.1.76

[B14] Lighthall N. R. Mather M. Gorlick M. A. (2009). Acute stress increases sex differences in risk seeking in the balloon analogue risk task. PloS ONE 47:e6002 10.1371/journal.pone.0006002 19568417 PMC2698212

[B15] Lighthall N. R. Sakaki M. Vasunilashorn S. Nga L. Somayajula S. Chen E. Y. (2011). Gender differences in reward-related decision processing under stress. Soc. Cogn. Affect. Neurosci. 7, 476–484 10.1093/scan/nsr026 21609968 PMC3324572

[B16] Mather M. Lighthall N. R. (2012). Risk and reward are processed differently in decisions made under stress. Curr. Dir. Psychol. Sci. 21, 36–41 10.1177/0963721411429452 22457564 PMC3312579

[B17] Nater U. M. Rohleder N. (2009). Salivary alpha-amylase as a non-invasive biomarker for the sympathetic nervous system: current state of research. Psychoneuroendocrinology 34, 486–496 10.1016/j.psyneuen.2009.01.014 19249160

[B18] Nater U. M. Rohlederc N. Schlotze W. Ehlert U. Kirschbaum C. (2007). Determinants of the diurnal course of salivary alpha-amylase. Psychoneuroendocrinology 32, 392–401 10.1016/j.psyneuen.2007.02.007 17418498

[B19] Newcombe V. F. J. Outtrim J. G. Chatfield D. A. Manktelow A. Hutchinson P. J. Coles J. P. (2011). Parcellating the neuroanatomical basis of impaired decision-making in traumatic brain injury. Brain 134, 759–768 10.1093/brain/awq388 21310727 PMC3044832

[B20] Pabst S. Brand M. Wolf O. T. (2013). Stress and decision-making: a few minutes make all the difference. Behav. Brain Res. 250, 39–45 10.1016/j.bbr.2013.04.046 23643690

[B21] Porcelli A. J. Delgado M. R. (2009). Acute stress modulates risk taking in financial decision making. Psychol. Sci. 20, 278–283 10.1111/j.1467-9280.2009.02288.x 19207694 PMC4882097

[B22] Porcelli A. J. Lewis A. H. Delgado M. R. (2012). Acute stress influences neural circuits of reward processing. Front. Neurosci. 6:157 10.3389/fnins.2012.00157 23125822 PMC3485541

[B23] Preston S. D. Buchanan T. W. Stansfield R. B. Bechara A. (2007). Effects of anticipatory stress on decision making in a gambling task. Behav. Neurosci. 121, 257–263 10.1037/0735-7044.121.2.257 17469915

[B24] Putman P. Antypa N. Crysovergi P. van der Does W. A. J. (2010). Exogenous cortisol acutely influences motivated decision making in healthy young men. Psychopharmacology 208, 257–263 10.1007/s00213-009-1725-y 19953227 PMC2797621

[B25] Rogers R. D. Everitt B. J. Baldacchino A. Blackshaw A. J. Swainson R. Wynne K. (1999). Dissociable deficits in the decision-making cognition of chronic amphetamine abusers, opiate abusers, patients with focal damage to prefrontal cortex, and tryptophan-depleted normal volunteers: evidence for monoaminergic mechanisms. Neuropsychopharmacology 20, 322–339 10.1016/S0893-133X(98)00091-8 10088133

[B26] Schwabe L. Hoeffken O. Tegenthoff M. Wolf O. T. (2013). Opposite effect of noradrenergic arousal on amygdala processing of fearful faces in men and women. Neuroimage 73, 1–7 10.1016/j.neuroimage.2013.01.057 23380165

[B27] Starcke K. Brand M. (2012). Decision making under stress: a selective review. Neurosci. Biobehav. Rev. 36, 1228–1248 10.1016/j.neubiorev.2012.02.003 22342781

[B28] Takahashi T. Ikeda K. Fukushima H. Hasegawa T. (2007). Salivary alpha-amylase and hyperbolic discounting in male humans. Neuroendocrinol. Lett. 28, 17–20 17277731

[B29] Taylor M. K. Sausen K. P. Mujica-Parodi L. R. Potterat E. G. Yanagi M. A. Kim H. (2007). Neurophysiologic methods to measure stress during survival, evasion, resistance, and escape training. Aviat. Space. Environ. Med. 78(5 Suppl.), B224–B230 17547323

[B30] Ter Horst J. P. Kentrop J. de Kloet E. R. Oitzl M. S. (2013). Stress and estrous cycle affect strategy but not performance of female C57BL/6J mice. Behav. Brain Res. 241, 92–95 10.1016/j.bbr.2012.11.040 23219839

[B31] Tschibelu E. Elman I. (2011). Gender differences in psychosocial stress and in its rela- tionship to gambling urges in individuals with pathological gambling. J. Addict. Dis. 30, 81–87 10.1080/10550887.2010.531671 21218314

[B32] van den Bos R. Davies W. Dellu-Hagedorn F. Goudriaan A. E. Granon S. Homberg J. (2013a). Cross-species approaches to pathological gambling: a review targeting sex differences, adolescent vulnerability and ecological validity of research tools. Neurosci. Biobehav. Rev. 37, 2454–2471 10.1016/j.neubiorev.2013.07.005 23867802

[B33] van den Bos R. Homberg J. de Visser L. (2013b). A critical review of sex differences in decision-making tasks, focus on the Iowa Gambling Task. Behav. Brain Res. 238, 95–108 10.1016/j.bbr.2012.10.002 23078950

[B34] van den Bos R. Jolles J. W. Homberg J. R. (2013c). Social modulation of decision-making: a cross-species review. Front. Hum. Neurosci. 7:301 10.3389/fnhum.2013.00301 23805092 PMC3693511

[B35] van den Bos R. de Visser L. van de Loo A. J. A. E. Mets M. A. J. van Willigenburg G. M. Homberg J. R. (2012). Sex differences in decision-making in adult normal volunteers are related to differences in the interaction of emotion and cognitive control, in Handbook on Psychology of Decision-making, eds Moore K. O. Gonzalez N. P. (Hauppage, NY: Nova Science Publisher Inc.), 179–198

[B36] van den Bos R. Harteveld M. Stoop H. (2009). Stress and decision-making in humans, performance is related to cortisol-reactivity, albeit differently in men and women. Psychoneuroendocrinology 34, 1449–1458 10.1016/j.psyneuen.2009.04.016 19497677

[B37] Wang J. Korczykowski M. Rao H. Fan Y. Pluta J. Gur R. C. (2007). Gender difference in neural response to psychological stress. Soc. Cogn. Affect. Neurosci. 2, 227–239 10.1093/scan/nsm018 17873968 PMC1974871

